# Off-label prescribing of tamsulosin: A nationwide retrospective study combining prescription and regulatory data

**DOI:** 10.1016/j.rcsop.2025.100679

**Published:** 2025-11-02

**Authors:** Anna Artner, Ákos Niedermüller, Máté Attila Csapó, Nikoletta Ngo Hanh, Emília Fekete, Romána Zelkó, Szilvia Sebők

**Affiliations:** a Center of Pharmacology and Drug Research & Development, Budapest, Hungary; b University Pharmacy - Department of Pharmacy Administration, Semmelweis University, Hőgyes Endre street 7, 1092 Budapest, Hungary

**Keywords:** Tamsulosin, Off-label use, Female patients, Prescription patterns, Regulatory compliance

## Abstract

**Background:**

Off-label use of medicines raises important safety and regulatory concerns. Tamsulosin, an alpha-1 adrenergic receptor blocker originally approved for benign prostatic hyperplasia (BPH) in men, is also prescribed beyond licensed indications.

**Aim:**

This study aimed to describe national on-label and off-label prescribing patterns of tamsulosin in Hungary using insurance and regulatory data.

**Methods:**

We conducted a retrospective, cross-sectional descriptive analysis of two national datasets: the National Health Insurance Fund (NEAK) database of reimbursed prescriptions (2019–2023) and the National Institute of Pharmacy and Nutrition (NNGYK) records of off-label authorisations (2009–2023). Prescriptions were analyzed by sex and International Classification of Diseases 10th Revision (ICD-10) codes.

**Results:**

A total of 906,011 prescriptions were linked to 214 ICD-10 codes; 805 entries lacked identifiable codes. Of these, 888,830 (98.1 %) were for men and 17,181 (1.9 %) for women. Female prescriptions most frequently carried codes for ureteral stones (2805; 16.3 %), dysuria (1872; 10.9 %), urinary retention (1722; 10.0 %), and kidney stones (1498; 8.7 %). Notably, 2845 prescriptions (16.6 %) in women were linked to prostate-related codes, suggesting coding errors. NNGYK approved eight individual off-label requests, mainly for urological indications.

**Conclusions:**

Off-label tamsulosin prescribing in Hungary is relatively infrequent but concentrated in women. These findings highlight the importance of pharmacovigilance, clinician awareness, and further research to clarify its therapeutic role in non-BPH indications.

## Highlights

• Tamsulosin prescriptions analyzed from 2019 to 2023 in Hungary
• 906,011 prescriptions associated with 214 different ICD codes
• Increasing trend of prescriptions for female patients was observed
• Off-label use in women: urinary retention, kidney stones, pelvic pain
• Further research needed for off-label uses; improved guidelines required

## 1 Introduction

Use of medications off-label—that is, outside the scope of their approved indications—is common in clinical practice and often arises when standard therapies are ineffective or unavailable, or when preliminary evidence from clinical trials suggests potential benefit.^1^ Physicians typically reserve off-label use for cases where licensed alternatives have been exhausted, given the ethical, clinical, and legal responsibilities involved.^2^

Tamsulosin, an alpha-1 adrenergic receptor antagonist, was developed by a Japanese pharmaceutical company during the late 1970s through 1980s. Early preclinical and clinical studies demonstrated its ability to relax smooth muscle in the prostate and bladder neck, leading to reduced urinary obstruction and symptom relief in men with benign prostatic hyperplasia (BPH).^3^^,^^4^ Following these results, tamsulosin was approved in Japan in 1993, in the United States in 1997, and in Europe later in the 1990s. Today, it is widely prescribed for lower urinary tract symptoms (LUTS) associated with BPH.

In Hungary, off-label drug use is regulated under the 2005 Act XCV on Medicinal Products for Human Use. Physicians must apply to the National Institute of Pharmacy and Nutrition (NNGYK) for approval before initiating off-label therapy.^5^ Each application is patient- and indication-specific, requiring detailed justification, including references to clinical trial results, published studies, or international guidelines supporting the proposed use. Importantly, approval is not automatic: physicians must demonstrate that conventional therapies are unsuitable or ineffective, and they must commit to reporting treatment outcomes and adverse events. This system reflects a structured approach designed to balance therapeutic innovation with patient safety and accountability.^6^

Despite these safeguards, real-world prescription data suggest that off-label use of tamsulosin may occur outside formal approval channels. Such patterns raise questions about the extent, justification, and implications of this practice.

The aim of this study was therefore to evaluate national prescribing trends for tamsulosin in Hungary, with particular attention to off-label indications and gender distribution, using data from the National Health Insurance Fund (NEAK).^7^ By mapping these trends, we sought to provide insights into current prescribing behaviour and to identify areas where further research and clinical guidance may be needed.

## 2 Materials and methods

### 2.1 Data collection

This retrospective, cross-sectional study utilized two main data sources: the NEAK database and the NNGYK website. These sources provided comprehensive insights into the prescription and off-label use of tamsulosin in Hungary:

1. NEAK: This dataset included aggregated prescription data for all reimbursed tamsulosin prescriptions issued during the study period january 1, 2019, to december 31, 2023. Information available included sex and the number of prescriptions associated to ICD-10 diagnostic codes year by year.
2. NNGYK: This dataset provided information on regulatory submissions and authorizations related to off-label use requests in Hungary. These data offered insight into how off-label tamsulosin prescribing is monitored and approved at the regulatory level.

### 2.2 Study population

From the NEAK dataset, all patients with at least one reimbursed tamsulosin prescription during the study period were included. Data were analyzed at the aggregated level; no individual patient identifiers were available or accessible. The NNGYK dataset was analyzed to identify whether formal requests for off-label tamsulosin use had been submitted and to contextualize the observed prescribing patterns. All reimbursed tamsulosin prescriptions during the study period were included in the analysis. Inclusion was comprehensive, not random or purposive, as the dataset covered the full national prescribing population.

### 2.3 Definitions

On-label use was defined as prescriptions associated with a diagnosis of BPH in men. Off-label use was defined as prescriptions in women, or in men without a BPH-related diagnostic code. Also off-label use was identified by prescriptions associated with ICD-10 diagnostic codes other BPH in men. Prescriptions linked to diagnoses with no established clinical rationale (e.g., malignant neoplasms of the bladder or prostate, cystitis) were considered “unsupported off-label use.”

### 2.4 Data analysis

Descriptive analyses were performed to characterize prescribing patterns. Results are reported as absolute numbers and percentages of prescriptions per diagnostic group, stratified by sex. Data from NEAK were analyzed using Microsoft Excel, and findings are presented in both tabular and narrative form. NNGYK data were used qualitatively to provide regulatory context and assess whether observed off-label indications corresponded to formally authorized uses.

### 2.5 Ethical considerations

The study did not involve identifiable patient data. Both datasets contained aggregated, anonymized information only. According to Hungarian regulations, no ethical approval was required. Access to NEAK and NNGYK data was granted through their respective official request procedures.

### 2.6 NEAK database analysis

Data on tamsulosin prescriptions were extracted from the NEAK database, covering the period from January 1, 2019, to December 31, 2023. The dataset included information about ICD-10 codes associated with prescriptions and the annual distribution of these prescriptions by gender. The obtained dataset did not include any personal data about the patients, preserving confidentiality while still enabling an analysis of prescription trends.

NEAK, as the official body managing Hungary's Health Insurance Fund, is responsible for the registration and reimbursement of drugs, as well as the oversight of funding and pricing processes, as defined by Government Decree 386/2016 (XII. 2.).^8^ Since NEAK records all subsidized prescriptions issued in Hungary, this dataset comprehensively reflects national trends in tamsulosin use.

The data collected were analyzed using descriptive statistical methods in Microsoft Excel. Visualizations, such as diagrams, were created to illustrate gender-specific prescription patterns. The analysis also explored the frequency of ICD-10 codes linked to tamsulosin prescriptions, with special attention to off-label usage.

### 2.7 Off-label use data

To investigate off-label use, data were retrieved from an Excel file available on the website of NNGYK.^9^ The dataset spanned from 2009 to 2023 and included all recorded requests for off-label use of tamsulosin. Search terms such as “tamsulosin,” “tamszulozin,” and “tamsulozin” were used to identify relevant entries.

The Excel table provided detailed information about each off-label use request, including the active ingredient, dosage form, strength, requested indication, patient demographics, and the decision made (approved or rejected). Additional details, such as the decision date, justification references, and authorization conditions, were also recorded.

Analysis of this dataset revealed eight relevant off-label use requests, the details of which are presented in Table 1.

**Table 1:** Tamsulosin off-label approvals between 2009 and 2023.

**Active Ingredient**	**Dosage Form and Strength**	**Requested Indication**	**Dosage**	**Patient**'s **Gender and Age**	**Decision (approved or rejected, with reasons for rejection if applicable)**	**Justification (literature references**)
tamsulosin	Retard hard capsule, 0,4 mg	persistent bladder neck dysfunction	1 × 0,1 mg-0,2 mg, continuous	male, 2 years old	approved	Based on literary references, the requested treatment can be supported.
tamsulosin	Retard hard capsule, 0,4 mg	persistent bladder neck dysfunction	1 × 0,1 mg-0,2 mg, continuous	male, 5 years old	approved	Based on literary references, the requested treatment can be supported.
tamsulosin	Retard hard capsule, 0,4 mg	persistent bladder neck dysfunction	1 × 0,1 mg-0,2 mg, continuous	male, 3 years old	approved	Based on literary references, the requested treatment can be supported.
tamsulosin	Modified-release capsule, 0,4 mg	persistent bladder neck dysfunction	1 × 0,1 mg-0,2 mg, continuous	male, 2 years old	approved	Based on literary references, the requested treatment can be supported.
tamsulosin	Modified-release capsule, 0,4 mg	persistent bladder neck dysfunction	1 × 0,1 mg-0,2 mg, continuous	male, 5 years old	approved	Based on literary references, the requested treatment can be supported.
tamsulosin	Modified-release capsule, 0,4 mg	persistent bladder neck dysfunction	1 × 0,1 mg-0,2 mg, continuous	male, 3 years old	approved	Based on literary references, the requested treatment can be supported.
tamsulosin	Retard hard capsule, 0,4 mg	childhood ureterolithiasis	tamsulosin: 1 caps/evening	female, 90 years old	approved	Request supported by literary data: Based on literature, the treatment may be effective for the requested condition.
tamsulosin	Retard hard capsule, 0,4 mg	kidney stones, ureteral stone	1 × 0,4 mg/day	female, 7 years old	approved	Request supported by literary data: Based on literature, the treatment may be effective for the requested condition.

All abbreviations used in this table are defined as follows: tamsulosin is an alpha-1 adrenergic receptor blocker; off-label approvals represent uses outside officially approved indications.

The study is reported in accordance with the STROBE (Strengthening the Reporting of Observational Studies in Epidemiology) guidelines.^10^

## 3 Results

**Fig. 1:**
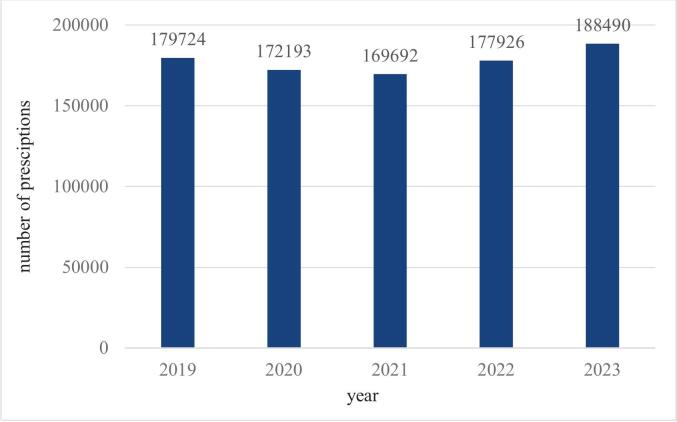
*a,* Tamsulosin prescriptions for male patients in Hungary between 2019 and 2023. *b,* Tamsulosin prescriptions for female patients in Hungary between 2019 and 2023.

Between 2019 and 2023, a total of 906,011 tamsulosin prescriptions were registered, associated with 214 different ICD-10 codes **(Appendix 1)**. Among these, 805 prescriptions lacked identifiable ICD-10 codes. Of the prescriptions with ICD-10 codes, 888025 (98,1 %) were issued for male patients, while 17,181 (1,9 %) were for female patients. The annual distribution of prescriptions is illustrated in Fig. 1. 2023 had the most prescriptions (192,309, which means 21,2 %), while 2021 had the least (172,642, 19,1 %). The most prescriptions for male patients occurred in 2023, for female patients in 2019.

**Fig. 2:**
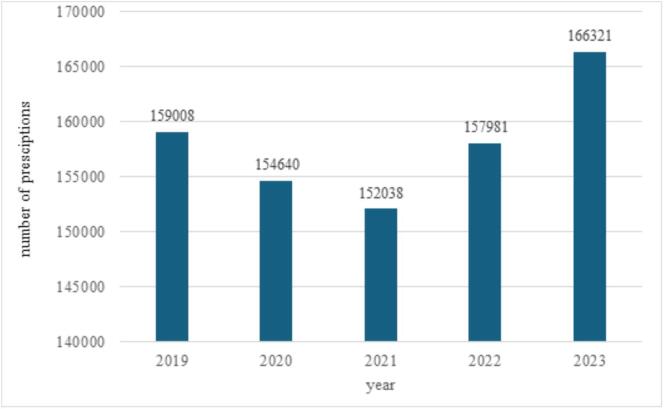
*a,* Number of tamsulosin prescriptions for male patients for prostate related ICD-10 codes (1B12.5; 2C82.Z; 2E67.5; 2F34; 2F97; GA90; GA91.Y; GA91.Z; GA91.0; GA91.1; GA91.2; GA91.3; GA91.4; GA91.5). *b,* Number of tamsulosin prescriptions for female patients for prostate related ICD-10 codes (1B12.5; 2C82.Z; 2E67.5; 2F34; 2F97; GA90; GA91.Y; GA91.Z; GA91.0; GA91.1; GA91.2; GA91.3; GA91.4; GA91.5). ICD-10: International Classification of Diseases10th Revision.

An analysis of the indications revealed that the prescription of tamsulosin-containing medications predominantly involved male patients, as shown in Fig. 2. 87,2 % of the prescriptions were for men with ICD codes associated with BPH and related LUTS, aligning with the official indications of the medication. However, such codes were also recorded for female patients **(**Fig. 2**)**, in 0,3 % of all prescriptions. This phenomenon may be partially explained by ICD codes being omitted by physicians and subsequently added during pharmacy dispensing.

**Fig. 3:**
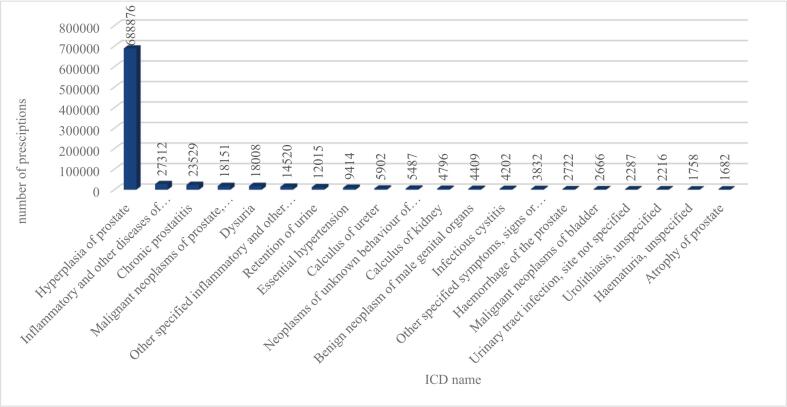
20 ICD-10 codes with the most tamsulosin prescriptions for male patients in Hungary between 2019 and 2023. Data represents annual prescription counts by gender from the National Health Insurance Fund database between 2019 and 2023.

**Fig. 4:**
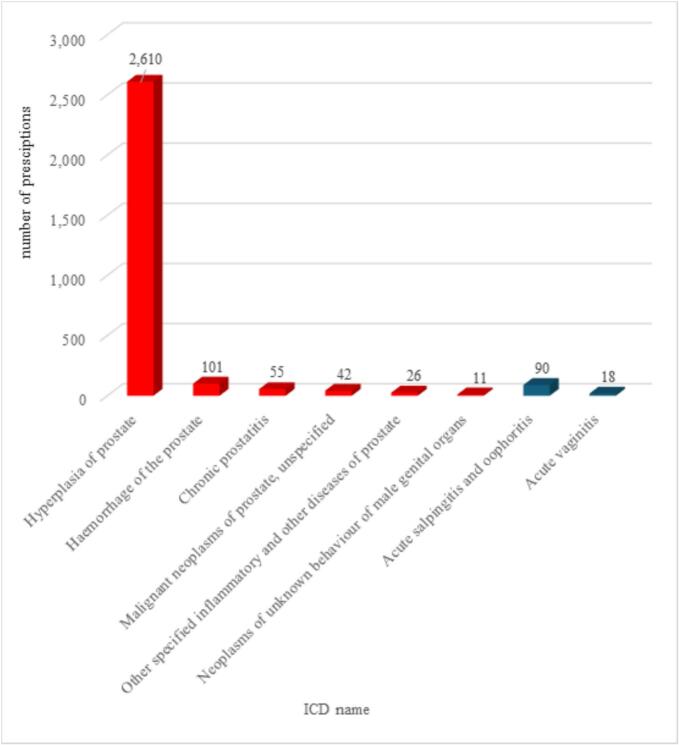
Tamsulosin prescriptions with irrelevant ICD-10 codes for female and male patients in Hungary between 2019 and 2023. Data represents annual prescription counts by gender from the National Health Insurance Fund database between 2019 and 2023.

Data from the NEAK database were analyzed to identify the primary indications for tamsulosin-containing medications between 2019 and 2023, broken down by gender. The findings are presented in Table 2, Table 3 **and** Fig. 3. Table 2 shows that the most frequent tamsulosin use for women in this period happened for kidney (8,7 % of all prescriptions for women) and ureteral stones (16,3 %), dysuria (10,9 %) and retention of urine (10 %). As it can be seen in Table 3 **and** Fig. 3, for male patients the most prescriptions were linked to hyperplasia and inflammation of prostate, (82,4 % of all prescriptions for men) and urinary disorders. Further data analysis revealed that irrelevant ICD codes were occasionally associated with tamsulosin prescriptions for both genders, as detailed in Fig. 4. Between 2019 and 2023 16,6 % of the prescriptions (2845 prescriptions) occurred for female patients with prostate-related ICD-10 codes. The irrelevant ICD-10 codes for male patients were vaginitis (0,01 %; 90) and oophoritis (0,002 %; 18). The possible explanation for these codes could be the above-mentioned omission by physicians, or their prescribing for the codes which are usually associated to tamsulosin.

**Table 2:** ICD-10 names with the most tamsulosin prescriptions for female patients in Hungary between 2019 and 2023.

ICD name	Number of prescriptions
Calculus of ureter	2805
Dysuria	1872
Retention of urine	1722
Calculus of kidney	1498
Urinary incontinence, unspecified	1112
Urolithiasis, unspecified	784
Infectious cystitis	612
Disorder of bladder, unspecified	398
Other specified disorders of bladder	299

ICD-10: International Classification of Diseases10th Revision; prescriptions refer to reimbursed tamsulosin prescriptions from the National Health Insurance Fund database.

**Table 3:** ICD-10 names with the most tamsulosin prescriptions for male patients in Hungary between 2019 and 2023.

ICD name	Number of prescriptions
Hyperplasia of prostate	688,876
Inflammatory and other diseases of prostate, unspecified	28,527
Chronic prostatitis	23,529
Malignant neoplasms of prostate, unspecified	18,151
Dysuria	18,008
Retention of urine	12,015
Other specified inflammatory and other diseases of prostate	14,520
Essential hypertension	9414
Calculus of ureter	5902
Neoplasms of unknown behaviour of male genital organs	5487
Calculus of kidney	4796
Benign neoplasm of male genital organs	4409
Infectious cystitis	4202
Other specified symptoms, signs or clinical findings involving the urinary system	3832
Haemorrhage of prostate	2722
Malignant neoplasms of bladder	2666
Urinary tract infection, site not specified	2287
Urolithiasis, unspecified	2216
Haematuria, unspecified	1758
Atrophy of prostate	1682
Urinary incontinence, unspecified	1552
Disorder of bladder, unspecified	1362
Prostatocystitis	1253
Other specified diseases of the genitourinary system	1126
Bladder neck obstruction	1112
Other specified nonfamilial nongentic cystic kidney disease	872
Neoplasms of unknown behaviour of urinary organs	748
Sexual functions	672
Cystitis	634
Chronic kidney disease	558
Calculus in bladder	516
Other specified disorders of bladder	318

ICD-10: International Classification of Diseases10th Revision; prescriptions refer to reimbursed tamsulosin prescriptions from the National Health Insurance Fund database.

Tamsulosin is primarily approved for managing LUTS associated with BPH. Consequently, most prescriptions for male patients are linked to ICD-10 codes related to BPH and its associated symptoms.

However, the analysis of prescription and dispensation data suggests that the range of ICD-10 codes associated with the use of tamsulosin is broader than the officially approved indications for the products available on the domestic market. Observations from Semmelweis University's community pharmacies revealed that tamsulosin is frequently prescribed for other urological conditions beyond its established therapeutic indications. Based on the findings, it becomes evident that tamsulosin has been prescribed not only for men but also for women. Our analysis focuses on the past five years of prescription data, as this period shows a noticeable increase in interest in tamsulosin among female patients. This trend is clearly reflected in the analyzed data.

## 4 Discussion

The findings of this study reveal notable trends in the off-label prescribing of tamsulosin in Hungary, particularly among female patients. While most prescriptions align with approved indications for BPH in men, a significant proportion—especially in women—are associated with indications not formally approved or supported by robust clinical evidence. These patterns raise important clinical, regulatory, and pharmacovigilance questions. In the following section, we interpret these findings in the context of international literature, discuss potential therapeutic justifications and safety concerns, and reflect on the broader implications for healthcare systems and regulatory oversight.

It is important to note that many of the ICD-10 codes listed in Table 2, Table 3 lack sufficient literature support, or only limited data are available regarding the safety and efficacy of tamsulosin. These data should be interpreted cautiously, particularly for indications such as cystitis or malignant neoplasms of the prostate and bladder. An unexpected increase in prescriptions associated with prostate-related codes in female patients was observed in 2019. As women cannot be diagnosed with prostate disease, this finding most likely reflects coding errors or changes in coding practice rather than genuine clinical prescribing trends. This highlights the importance of cautious interpretation of administrative coding data in pharmacoepidemiological studies. Specific studies or trials validating the efficacy of tamsulosin in treating these diseases are absent. For example, the treatment of bladder cancer typically involves chemotherapy, immunotherapy, or surgical interventions and does not include the use of alpha-1 receptor blockers. Thus, the role of tamsulosin in such indications is not substantiated, and its use should be approached with caution.^11^

According to the literature, tamsulosin is most commonly used off-label in women for treating urinary stones, dysuria, kidney stones, and incontinence.^12^, ^13^, ^14^

LUTS, interstitial cystitis, and menopause-related issues significantly impact women's quality of life, necessitating the exploration of new treatment options. Studies have demonstrated that tamsulosin effectively reduces voiding difficulties and the associated pain in female patients.^12^ Additionally, Maiti et al. provide new perspectives on the drug's potential to alleviate menopausal symptoms, further broadening its applicability.^13^

The primary advantage of tamsulosin lies in its selective action on alpha-1 A receptors in the female urinary system, which causes less frequency of undesirable side effects such as dizziness or hypotension compared to nonselective alpha-blockers.^15^ While generally well-tolerated, potential side effects, including retrograde ejaculation and an increased risk of urinary tract infections, require attention.^3^ For female patients, understanding the drug's side effects and mechanism of action is particularly critical, as anatomical and hormonal differences influence treatment decisions. Educating patients and closely monitoring their treatment are essential components of clinical practice.

The interpretation of our findings requires consideration of tamsulosin's pharmacological profile and its established as well as potential clinical applications. Tamsulosin is a selective alpha-1 A-adrenergic receptor antagonist that relaxes smooth muscle in the prostate and bladder neck, thereby improving urinary flow.^3^^,^^4^ Its selectivity reduces the risk of systemic adverse effects such as dizziness and orthostatic hypotension compared to non-selective alpha-blockers.^16^

While originally developed for men with lower urinary tract symptoms (LUTS) due to benign prostatic hyperplasia (BPH), several studies have investigated its use in other urological contexts. In women, tamsulosin has been studied for urinary retention, voiding dysfunction, and kidney stone passage.^12^^,^^13^^,^^17^^,^^18^ Evidence suggests improvements in voiding difficulty and pain, as well as potential benefits in peri- and postmenopausal patients.^13^ Nonetheless, adverse effects, including retrograde ejaculation, dizziness, and an increased risk of urinary tract infections, must be considered.^19^

Importantly, some ICD-10 codes in our dataset (e.g., bladder cancer) are not supported by clinical evidence. Standard treatment for bladder cancer involves surgery, chemotherapy, or immunotherapy, and no role for alpha-blockers has been established.^11^ This highlights the need for caution in interpreting off-label prescribing patterns that may not have a pharmacological rationale.

Taken together, these pharmacological insights provide context for our results: while tamsulosin's mechanism supports its use in selected female urinary disorders, robust evidence is still limited. Future prospective clinical trials are required to clarify its safety and effectiveness in these off-label applications.

International data reflect similar trends. In Japan and Thailand, tamsulosin has been explored in female patients with LUTS, with studies reporting symptomatic improvement and enhanced quality of life.^12^^,^^18^ A systematic review by Zhang et al. supports the use of tamsulosin for voiding dysfunction in women, while emphasizing the need for randomized controlled trials to confirm efficacy and safety.^18^ In India, a comparative study suggested that tamsulosin may be effective in perimenopausal women with LUTS, potentially offering an alternative to hormonal treatments.^13^ Meanwhile, in the United States, off-label tamsulosin use in emergency departments for urolithiasis remains common practice, despite ongoing controversy over its benefit in smaller ureteral stones.^17^^,^^20^

While these findings suggest promising therapeutic avenues, off-label use must be approached with caution. Tamsulosin, although generally well-tolerated, can lead to adverse drug reactions (ADRs) such as dizziness, orthostatic hypotension, fatigue, and retrograde ejaculation—some of which have been reported in female patients as well.^11^^,^^19^ Furthermore, drug–drug interactions, particularly involving cytochrome P450 3A4 (CYP3A4) inhibitors or other antihypertensives, may enhance the risk of hypotension and falls. Case reports have highlighted such risks, including in women, reinforcing the need for vigilance in off-label prescribing.^21^

Our findings also raise concerns regarding the adequacy of regulatory oversight. Although Hungarian law mandates prior approval by NNGYK for any off-label use, the NEAK database shows that thousands of tamsulosin prescriptions are associated with ICD-10 codes outside the drug's approved indications, including female-specific conditions. Yet only a handful of official off-label approvals were issued by the NNGYK between 2009 and 2023. This discrepancy implies either a lack of enforcement, widespread non-compliance, or gaps in the approval infrastructure, each of which warrants further investigation.

In addition to clinical and regulatory implications, the frequent off-label use of tamsulosin may present operational challenges. Pharmacists and outpatient providers often face difficulty evaluating the appropriateness of off-label prescriptions, particularly when ICD-10 coding errors or missing indication data obscure the clinical rationale. Without standardized guidelines or adequate information, healthcare providers may struggle to support safe medication use. This may also strain the pharmacist–physician–patient communication dynamic and increase the risk of medication errors.

Overall, tamsulosin has demonstrated effectiveness in alleviating urinary dysfunction symptoms; however, safety concerns and potential side effects remain important considerations in treatment planning, particularly for female patients.^14^ Real-world data, such as those presented in this study, can assist regulatory bodies and clinicians in identifying high-risk off-label trends. Targeted pharmacovigilance efforts and national guidance on selected off-label indications may help narrow the gap between everyday clinical practice and evidence-based prescribing. At the same time, the drivers of off-label tamsulosin use in practice remain unclear. Input from urology specialists could provide valuable insight into clinical decision-making and perceived therapeutic needs, while future prospective studies are warranted to further evaluate the rationale, effectiveness, and safety of such prescribing patterns.

## 5 Study strengths and limitations

### 5.1 Strengths

This study provides several important contributions to understanding tamsulosin utilization patterns. First, it represents a comprehensive population-based analysis utilizing national-level administrative data covering the entire Hungarian healthcare system over a five-year period (2019–2023). The large sample size and complete population coverage minimize selection bias and provide robust estimates of prescribing patterns.

Second, our inclusion of both male and female patients across all age groups provides valuable insights into off-label prescribing practices, particularly the emerging use of tamsulosin in women for urological conditions. Third, the integration of NEAK prescription data with NNGYK regulatory approval records enables accurate classification of off-label versus approved uses based on Hungarian regulatory framework.

Fourth, the real-world evidence approach captures actual clinical practice patterns rather than controlled clinical trial conditions, providing insights into how tamsulosin is prescribed in routine healthcare delivery. Fifth, our systematic deduplication methodology ensures accurate representation of prescribing events and prevents inflation of utilization estimates.

### 5.2 Limitations

This study has several limitations that should be considered when interpreting the findings. First, the analysis relied on prescription data from the National Health Insurance Fund database, which does not provide clinical confirmation of diagnoses. Consequently, we cannot verify whether the ICD-10 codes associated with prescriptions accurately reflected the patients' conditions. Second, potential coding bias or errors may have influenced the categorization of prescriptions, as demonstrated by the presence of clinically irrelevant ICD-10 codes in both male and female patients. Third, the dataset did not allow assessment of the duration of treatment or adherence patterns, which limits conclusions about long-term use of tamsulosin in off-label contexts. Fourth, the data did not include information on the prescribing physician, care setting, or clinical rationale, which would have provided important context for understanding prescribing behaviour. Finally, as with all retrospective database studies, the findings are descriptive in nature and cannot establish causal relationships. Because the NEAK dataset did not contain clinical outcome information, we were unable to determine whether the intended therapeutic effect of off-label tamsulosin prescribing was achieved.

## 6 Conclusions

This study shows that while most tamsulosin prescriptions in Hungary are issued for approved indications in men, a considerable proportion are written for women and other off-label conditions, often without strong supporting evidence. Such prescribing patterns underline both the potential therapeutic opportunities and the safety challenges of off-label use.

To improve practice, prospective clinical trials are essential to establish the efficacy and safety of tamsulosin in female patients and non-BPH indications. In addition, training and educational programs for prescribers and pharmacists would strengthen awareness of appropriate off-label use and support patient safety.

By combining evidence generation with professional education, the therapeutic potential of tamsulosin can be explored responsibly while minimizing risks for patients.

## Funding

The research was not supported by any specific funding.

## CRediT authorship contribution statement

**Anna Artner:** Writing – original draft, Visualization, Methodology, Investigation, Formal analysis, Data curation, Conceptualization. **Ákos Niedermüller:** Writing – original draft, Visualization, Methodology, Investigation, Formal analysis, Data curation, Conceptualization. **Máté Attila Csapó:** Writing – original draft, Methodology, Investigation, Formal analysis, Data curation, Conceptualization. **Nikoletta Ngo Hanh:** Writing – original draft, Methodology, Investigation, Formal analysis, Data curation, Conceptualization. **Emília Fekete:** Writing – original draft, Methodology, Investigation, Formal analysis, Data curation, Conceptualization. **Romána Zelkó:** Writing – review & editing, Supervision, Conceptualization. **Szilvia Sebők:** Writing – review & editing, Supervision, Data curation, Conceptualization.

## Declaration of competing interest

The authors declare that they have no conflicts of interest. Furthermore, they affirm that there are no financial interests or personal relationships that could have influenced the findings or conclusions presented in this paper.

## Figures and Tables

**Table:** 

1B12.5	Tuberculosis of the genitourinary system
1B40	Acute rheumatic fever without mention of heart involvement
1D9Z	Unspecified viral infection of unspecified site
2A60	Acute myeloid leukaemias and related precursor neoplasms
2A83.1	Plasma cell myeloma
2B90.Z	Malignant neoplasms of colon, unspecified
2B92	Malignant neoplasms of rectum
2C25.Z	Malignant neoplasms of bronchus or lung, unspecified
2C80.Z	Malignant neoplasms of testis, unspecified
2C82.Z	Malignant neoplasms of prostate, unspecified
2C90	Malignant neoplasms of kidney, except renal pelvis
2C94	Malignant neoplasms of bladder
2C94·Y	Other specified malignant neoplasms of bladder
2C94.Z	Malignant neoplasms of bladder, unspecified
2E03	Malignant neoplasm metastasis in bone or bone marrow
2E67.5	High grade intraepithelial lesion of prostate
2F34	Benign neoplasm of male genital organs
2F35	Benign neoplasm of male urinary organs
2F97	Neoplasms of unknown behaviour of male genital organs
2F98	Neoplasms of unknown behaviour of urinary organs
3A00.Z	Iron deficiency anaemia, unspecified
3A71.Z	Anaemia due to chronic disease, unspecified
4A8Z	Allergic or hypersensitivity conditions of unspecified type
5A00	Hypothyroidism
5A0Z	Disorders of the thyroid gland or thyroid hormones system, unspecified
5A10	Type 1 diabetes mellitus
5A11	Type 2 diabetes mellitus
5A14	Diabetes mellitus, type unspecified
5A81.1	Testicular hypofunction
5B57.Z	Vitamin D deficiency, unspecified
5B5K.3	Iodine deficiency
5B7Z	Unspecified undernutrition
5C55·Y	Other specified inborn errors of purine, pyrimidine or nucleotide metabolism
5C77	Hypokalaemia
5C80	Hyperlipoproteinaemia
5C80.0	Hypercholesterolaemia
5C80.2	Mixed hyperlipidaemia
5C8Y	Other specified disorders of lipoprotein metabolism or lipidaemias
6A70.1	Single episode depressive disorder, moderate, without psychotic symptoms
6A73	Mixed depressive and anxiety disorder
6B0Z	Anxiety or fear-related disorders, unspecified
6E62	Secondary mood syndrome
7B2Z	Sleep-wake disorders, unspecified
8A00.0	Parkinson's disease
8A40	Multiple sclerosis
8A60.Z	Epilepsy due to unspecified structural or metabolic condition or disease
8B10·Y	Other specified transient ischaemic attack
8B10.Z	Transient ischaemic attack, unspecified
8B11.1	Cerebral ischaemic stroke due to intracranial large artery atherosclerosis
8B1Z	Cerebral ischaemia, unspecified
8B2Z	Cerebrovascular diseases, unspecified
8B93·Y	Other specified radiculopathy
8C03.0	Diabetic polyneuropathy
9C60	Glaucoma suspect
9C61.0	Primary open-angle glaucoma
BA00	Essential hypertension
BA00.Z	Essential hypertension, unspecified
BA03	Hypertensive crisis
BA04.Y	Other specified secondary hypertension
BA40.0	Unstable angina
BA40.Y	Other specified angina pectoris
BA40.Z	Angina pectoris, unspecified
BA4Z	Acute ischaemic heart disease, unspecified
BA50	Old myocardial infarction
BA51	Ischaemic cardiomyopathy
BA52	Coronary atherosclerosis
BA5Y	Other specified chronic ischaemic heart disease
BA5Z	Chronic ischaemic heart disease, unspecified
BB70	Aortic valve stenosis
BC42.1	Infectious myocarditis
BC43.0	Dilated cardiomyopathy
BC81.3	Atrial fibrillation
BC9Z	Cardiac arrhythmia, unspecified
BD1Y	Other specified heart failure
BD1Z	Heart failure, unspecified
BD40·Y	Other specified atherosclerotic chronic arterial occlusive disease
BD40.Z	Atherosclerotic chronic arterial occlusive disease, unspecified
BD52.2	Stricture of artery
BD55	Asymptomatic stenosis of intracranial or extracranial artery
BD56	Asymptomatic occlusion of intracranial or extracranial artery
BD70.1	Superficial thrombophlebitis of upper limbs
BD75·Y	Venous varicosities of other specified sites
CA02	Acute pharyngitis
CA07.0	Acute upper respiratory infection, site unspecified
CA08.0	Allergic rhinitis
CA20.Y	Other specified bronchitis
CA22.Z	Chronic obstructive pulmonary disease, unspecified
CA23.0	Allergic asthma
CA23.3	Unspecified asthma
CA40.Z	Pneumonia, organism unspecified
CA42.Z	Acute bronchitis, unspecified
DA06.0	Inflammatory conditions of jaws
DA22.0	Non-erosive gastro-oesophageal reflux disease
DA22.1	Erosive gastro-oesophageal reflux disease
DA25	Oesophageal ulcer
DA42	Gastritis
DA42.Z	Gastritis, unspecified
DB60.Z	Haemorrhoids, unspecified
DD51	Inguinal hernia
EA8Z	Dermatitis or eczema, unspecified
EA90.0	Plaque psoriasis
FA00.Z	Osteoarthritis of hip, unspecified
FA01.Z	Osteoarthritis of knee, unspecified
FA25.0	Primary gout
FA25.2	Gout without specification whether primary or secondary
FA26.1	Hydroxyapatite deposition disease
FA70.0	Kyphosis
FA70.1	Scoliosis
FA8Z	Degenerative condition of spine, unspecified
FB82.1	Osteochondrosis or osteochondritis dissecans
FB83.1Z	Osteoporosis, unspecified
GA02.0	Acute vaginitis
GA07.0	Acute salpingitis and oophoritis
GA13.1	Low grade squamous intraepithelial lesion of vulva
GA90	Hyperplasia of prostate
GA91.0	Chronic prostatitis
GA91.1	Abscess of prostate
GA91.2	Prostatocystitis
GA91.3	Calculus of prostate
GA91.4	Haemorrhage of the prostate
GA91.5	Atrophy of prostate
GA91.Y	Other specified inflammatory and other diseases of prostate
GA91.Z	Inflammatory and other diseases of prostate, unspecified
GB00.0	Encysted hydrocele
GB00.2	Spermatocele
GB00·Y	Other specified hydrocele or spermatocele
GB00.Z	Hydrocele or spermatocele, unspecified
GB01.0	Torsion of testis
GB02.0	Orchitis, epididymitis or epididymo-orchitis with abscess
GB02.1	Orchitis, epididymitis or epididymo-orchitis without abscess
GB03	Atrophy of testis
GB04	Male infertility
GB05	Redundant prepuce, phimosis or paraphimosis
GB05.2	Phimosis
GB06.0	Balanoposthitis
GB06·Y	Other specified disorders of penis
GB07.0	Inflammatory disorders of seminal vesicle
GB0Y	Other specified diseases of the male genital system
GB0Z	Diseases of the male genital system, unspecified
GB20.0	Fibrocystic change of breast
GB40	Nephritic syndrome
GB41	Nephrotic syndrome
GB4Y	Other specified glomerular diseases
GB50	Acute tubulo-interstitial nephritis
GB55.Z	Chronic tubulo-interstitial nephritis, unspecified
GB56	Obstructive or reflux nephropathy
GB56.2	Hydronephrosis with ureteral orifice obstruction
GB56.4	Other or unspecified hydronephrosis
GB60.2	Acute kidney failure, stage 3
GB60.Z	Acute kidney failure, stage unspecified
GB61	Chronic kidney disease
GB6Z	Kidney failure, unspecified
GB70.0	Calculus of kidney
GB70.1	Calculus of ureter
GB71.0	Calculus in bladder
GB71.Z	Calculus of lower urinary tract, unspecified
GB7Z	Urolithiasis, unspecified
GB80·Y	Other specified nonfamilial nongenetic cystic kidney disease
GB8Z	Cystic or dysplastic kidney disease, unspecified
GB90	Certain specified disorders of kidney or ureter
GC00	Cystitis
GC00.1	Infectious cystitis
GC00.3	Interstitial cystitis
GC00·Y	Other specified cystitis
GC00.Z	Cystitis, unspecified
GC01.0	Bladder neck obstruction
GC01.2	Diverticulum of bladder
GC01.4	Neuromuscular dysfunction of bladder, not elsewhere classified
GC01·Y	Other specified disorders of bladder
GC01.Z	Disorder of bladder, unspecified
GC03	Urethral stricture
GC08	Urinary tract infection, site not specified
GC2Z	Diseases of the urinary system, unspecified
GC40.0Z	Prolapse of anterior vaginal wall, unspecified
GC8Y	Other specified diseases of the genitourinary system
HA01.1Z	Male erectile dysfunction, unspecified
MA15.0	Bacteraemia
MA3Y	Other specified symptoms, signs or clinical findings of blood, blood-forming organs, or the immune system
MB48.0	Vertigo
MB4D	Headache, not elsewhere classified
MC81.0	Tachycardia, unspecified
MD81.12	Pain localised to other parts of lower abdomen
MD81.4	Other and unspecified abdominal pain
MD92	Dyspepsia
ME82	Pain in joint
ME84.20	Lumbago with sciatica
ME84.2Z	Low back pain, unspecified
MF40·Y	Other specified problems of male genital organs
MF50.20	Stress incontinence
MF50.2Y	Other specified urinary incontinence
MF50.2Z	Urinary incontinence, unspecified
MF50.3	Retention of urine
MF50.4Z	Haematuria, unspecified
MF50.7	Dysuria
MF50·Y	Other specified aICDrmal micturition
MF5Y	Other specified symptoms, signs or clinical findings involving the urinary system
MF9Y	Other specified clinical findings on examination of urine, without diagnosis
MG3Z	Pain, unspecified
MG48	Unknown and unspecified causes of morbidity
MH2Y	Other specified symptoms, signs or clinical findings, not elsewhere classified
PB55·Y	Other specified unintentional exposure to explosion
QA00.0	General adult medical examination
QA02	Medical observation or evaluation for suspected diseases or conditions, ruled out
QA0A	Special screening examination for other diseases or disorders
QA0Z	Examination or investigation, unspecified
QA1C	Person with feared complaint in whom no diagnosis is made
QB4Z	Dependence on unspecified machine or device
QB63.0	Presence of transplanted kidney
QB92	Contact with health services for issue of repeat prescription
QC02.Z	Need for immunization against certain specified single infectious diseases, unspecified
QC05	Need for certain specified other prophylactic measures
QC0Z	Contact with health services related to immunizations or certain other prophylactic measures, unspecified
VV51	Sexual functions
VV5Y	Other specified genitourinary and reproductive functions
